# The Surgical Treatment of Septic Thrombosis and Other Complications of the Middle Ear Disease.—II

**Published:** 1892-01-09

**Authors:** 


### AURAL SURGERY.

### THE SURGICAL TREATMENT OF SEPTIC THROMBOSIS AND OTHER COMPLICATIONS OF THE MIDDLE EAR DISEASE.—II.

In a previous paper we discussed the diagnosis and considered the general principles of treatment in septic thrombosis of the lateral sinus. It remains for us to describe more in detail the method of procedure.

And first let us deal with cases in which our diagnosis is certain. Here we can at once explore the sinus. And this can best be done by a three-quarter inch trephine, the pin of which is placed at a point one inch behind the centre of the bony meatus, and on the level of its upper border. This is the position given by Ballance, and it is also the one given by Birmingham as a certain guide to the sinus, as the result of his numerous observations.* When the sinus is exposed, we must puncture it with a fine hollow needle to ascertain if it is plugged. If so, it would be unwise to disturb the clot before we had ligatured the vein in the neck, lest portions of the clot should be detached and form septic emboli. The vein should be ligatured in two places and divided between the ligatures, and the upper end then stitched to the upper angle of the wound, so as to facilitate drainage from the sinus. This has been done just below the level of the upper border of the thyroid.

When we have cut off the passage of septic clots in this way, then we may again deal with the sinus. It must be slit open as far as our trephine wound will allow, and the clot scooped away and syringed out with 1 in 2,000 perchloride solution; and if we have stitched the upper end of the divided jugular into the wound, we can open it, and syringe right through from the sinus.

If there is any reed to control the sinus, it must be done by pressure of artery forceps, or by plugging, never by the passing of a suture around, as this would open the intradural space and expose it to the risk of infection.

When we have in this way dealt with the sinus and vein, it is desirable to remove more bone in a forward direction, until the antrum is opened, so that we can thoroughly clean that out and have free drainage through the middle ear; and in operating for thrombosed sinus, we may often find an extra dural abscess in the groove for the sinus, and thus be able to evacuate this at the same time; and even if we do not find pus here, we must not be content until with a bent probe we have explored under the dura mater, over the tympanum, for an extra dura abscess is often present in this situation.

So much then for cases in which we have little or no doubt. The sinus is thrombosed: but how are we to proceed when in doubt?

The complications of middle ear disease which often cannot be distinguished at their onset are, septic thrombosis of the sinus, subdural abscess, early meningitis, and even pent-up pus in the middle ear. It is often quite impossible during the first few days to make a diagnosis between them. Vomiting, headache, shivering with rise in the temperature several degrees, and more or less drowsiness, are common to them all. Optic neuritis may or may not be present in any one of them. Continued rigors, or an irregular temperature, indicating pyæmia would not help us in our diagnosis, because this condition might be due to several complications; to a septic thrombosis in the sinus, subdural abscess, or merely to pent-up pus in the middle ear. We must look for the the more distinctive signs of thrombosis—signs of extension of clot already described in our previous paper, and the tenderness behind the mastoid, but septic thrombosis of the lateral sinus may exist, and yet there may be no signs of extension of clot, nor even the less reliable one of tenderness along the posterior border of the mastoid. Subdural abscess, again, may be present without any distinctive signs.

What are we to do, then, if we cannot make a diagnosis at the early stage? Are we to wait until more definite signs of thrombosis of the sinus or the coma of meningitis come on, before we do anything for the patient, because we cannot make an exact diagnosis earlier? No; we must try the simpler and less severe surgical methods, and if these fail, then we must explore further. If a person with acute or chronic ear disease begins to vomit, to complain of headache

* *Dublin Journal of Medical Science*, February, 1891.

to shiver, and to get drowsy, our first care must be to see that there is free discharge from the middle ear. If there is a polypus blocking the canal it must be removed; if the opening in the membrane does not seem free enough it must be enlarged, and the middle ear frequently and thoroughly syringed out. If the symptoms are not relieved in this way, we must be prepared to explore the interior of the mastoid. We should examine the mastoid vein, and if we find that thrombosed, then we may feel sure the lateral sinus is, and explore that first, and open up the mastoid antrum at the same time, so as to provide more perfect drainage of the middle ear. But unless we find the mastoid vein plugged, we must first explore the mastoid cells, and syringe from them through into the tympanum, and see if with this more thorough drainage the symptoms subside. If they do not we must expose the lateral sinus groove in the way already described, and see if an extra dural abscess exists here, or if the sinus is thrombosed. If we cannot explore the roof of the tympanum from here by means of the bent probe, we must trephine a short inch vertically above the centre of the meatus, so as to come down on the upper part of the petrous bone, where we can explore over the tympanic roof under the dura mater. To reach the antrum, we must perforate as near as possible to the posterior border of the meatus, and as high as its upper border, but not higher. Birmingham has shown by numerous anatomical observations* that this is the best and safest spot. The trephine (if we use one) ought not to be more than one-fourth of an inch in diameter, and should go straight in, but never for more than three-fourths of an inch lest the internal ear be damaged. There is risk in using a larger trephine for the deeper parts of wounding the sinus, which occasionally comes very near the meatus and near the surface. A small gouge is a better instrument.

* *Dublin Journal of Medical Science*, Feb., 1891.

